# CDK4/MERCs/PINK1 Axis Drives PFOA/HFPO‐TA‐Induced Cardiac Senescence via Mitophagy Defect and cGAS‐STING Activation: In Vitro Amelioration by Cycloastragenol

**DOI:** 10.1002/advs.77280

**Published:** 2026-08-18

**Authors:** Nuo‐Wa Li, Jun‐Ze Jiang, Ying‐Ying Liu, Tian‐Tian Zhang, Kanwar Kumar Malhi, Xin Yao, Jin‐Long Li

**Affiliations:** ^1^ College of Veterinary Medicine Northeast Agricultural University Harbin People's Republic of China; ^2^ Key Laboratory of Diagnosis and Treatment for Epizootic Diseases of Animals in Cold Regions of Heilongjiang Province Northeast Agricultural University Harbin People's Republic of China; ^3^ Key Laboratory of the Provincial Education Department of Heilongjiang for Common Animal Disease Prevention and Treatment Northeast Agricultural University Harbin People's Republic of China

**Keywords:** cardiotoxicity, CDK4, Cycloastragenol, HFPO‐TA, mitophagy, PFOA

## Abstract

Per‐ and polyfluoroalkyl substances (PFAS) are ubiquitous persistent environmental pollutants with high bioaccumulation potential and widespread human exposure risks in contaminated water, soil, and biota. Although extensive studies have established the hepatotoxic potential of PFAS, much less is known regarding their cardiotoxicity and the underlying molecular mechanisms. Here, we investigated the cardiotoxic effects and molecular pathways of two representative PFAS, perfluorooctanoic acid (PFOA) and its alternative hexafluoropropylene oxide trimer acid (HFPO‐TA). Exposure to either PFOA or HFPO‑TA caused cardiac structural and functional damage, mitochondrial morphological abnormalities, and increased cardiac senescence, with HFPO‐TA eliciting greater cardiotoxicity. Mechanistically, PFOA and HFPO‐TA suppress CDK4 expression, destabilize mitochondria‐endoplasmic reticulum contacts (MERCs), and impair PINK1/Parkin‐mediated mitophagy. This defective mitophagy promotes mitochondrial DNA leakage and subsequent activation of the cGAS‐STING pathway, ultimately driving cardiac senescence. Notably, cycloastragenol (CAG) effectively reverses CDK4 downregulation, restores MERCs stability, and attenuates PFOA/HFPO‐TA‐induced cardiac senescence in vitro. Collectively, this study uncovers a CDK4/MERCs/PINK1 axis mediating PFOA/HFPO‐TA‐induced myocardial toxicity and identifies CAG as a promising natural product for mitigating environmental pollutant‐induced cardiac senescence, offering insights for PFAS health risk assessment and intervention strategies.

## Graphical abstract

The present study demonstrated that PFOA and HFPO‐TA exposure suppressed CDK4, disrupted MERCs and impaired PINK1/Parkin‐mediated mitophagy, thereby accelerating cardiac senescence, whereas CAG effectively reversed these pathological changes in vitro. Our findings identify CDK4 as a critical regulator bridging mitophagy defects and cardiac senescence and highlight CAG as a promising therapeutic candidate for mitigating PFAS‐induced cardiotoxicity.

## 1 Introduction

Per‐ and polyfluoroalkyl substances (PFAS) comprise a large family of synthetic organofluorine compounds characterized by exceptional hydrophobicity and oleophobicity. These properties have led to their extensive application in diverse industrial and consumer products, including food contact materials, textile treatments, pharmaceutical manufacturing, firefighting foams, and aerospace and construction components [1, 2]. However, these same physicochemical characteristics confer extreme environmental persistence and pronounced bioaccumulation, prompting global regulatory attention [3, 4]. Among the > 4,700 PFAS congeners, perfluorooctanoic acid (PFOA) is the most ubiquitous legacy compound, with concentrations in contaminated soils reaching hundreds of µg/kg and in groundwater up to tens of µg/L. Although PFOA was added to the Stockholm Convention list, its hydrolytic half‐life exceeds 97 years, and continuous detection in wildlife and humans underscores its enduring presence [5, 6, 7]. A substantial body of literature has documented PFOA‐induced multi‐organ toxicity, including hepatic steatosis, reproductive impairment, and cardiovascular damage [8, 9], with PPARα as a canonical molecular target [10, 11, 12, 13]. Hexafluoropropylene oxide trimer acid (HFPO‐TA), the dominant industrial alternative for PFOA, has been reported to induce milder hepatotoxicity than PFOA, but emerging evidence indicates its higher potency in inducing hepatic metabolic disturbance and reproductive toxicity due to increased hepatic and serum accumulation [14, 15, 16, 17]. Nevertheless, the cardiovascular toxicity of HFPO‐TA in mammalian systems remains essentially uncharted, and the molecular mechanisms underlying PFAS‐induced cardiac damage are still fragmentary. This knowledge gap severely hinders the comprehensive environmental health risk assessment of PFAS and their alternatives.

Due to its exceptionally high energy requirements, the heart relies heavily on mitochondrial ATP synthesis and on robust quality‐control pathways, including mitophagy, to preserve normal function [18, 19]. PTEN‐induced kinase 1 (PINK1)/Parkin‐mediated mitophagy eliminates dysfunctional mitochondria, curbs reactive oxygen species (ROS) and protects against tissue injury [20]. When mitophagy is compromised, damaged mitochondria progressively accumulate, accompanied by disruption of cristae architecture and subsequent egress of mitochondrial DNA (mtDNA) into the cytoplasmic compartment. Once released into the cytosol, mtDNA initiates signaling through cGAS and its downstream adaptor, stimulator of interferon genes (STING) [21]. Recent work has uncovered a cGAS‐STING‐PERK‐eIF2α pathway that drives cellular senescence, an irreversible proliferative arrest characterized by senescence‐associated β‐galactosidase (SA‐β‐gal) activity and the senescence‐associated secretory phenotype (SASP) [22, 23]. Mitochondria‐endoplasmic reticulum contacts (MERCs) are key subcellular structures that spatially organize PINK1 recruitment to damaged mitochondria. Disruption of MERCs impairs PINK1 localization and thereby suppresses mitophagy. Nevertheless, whether MERCs dysfunction contributes to PFAS‐evoked impairment of cardiac mitophagy and subsequent senescence remains unexplored [24].

Cyclin‐dependent kinase 4 (CDK4) drives the G1→S transition of the cell cycle and is negatively controlled by the senescence‐associated proteins p16 and p21 [25, 26]. Emerging studies have revealed non‐cell‐cycle functions of CDK4, which localizes at MERCs, regulates MERCs structural stability and phosphorylates PKA substrates. CDK4 inactivation disrupts MERCs [27], impairs PINK1 recruitment to depolarized mitochondria, and inhibits PINK1/Parkin‐mediated mitophagy. These findings position CDK4 as a key hub connecting cellular senescence and organelle quality control, but its role in environmental pollutant‐induced cardiac senescence remains unexplored. In the past decade, bioactive compounds derived from traditional Chinese medicine have demonstrated considerable therapeutic promise [28, 29]. Within this context, *Astragalus membranaceus* (Huangqi) occupies a prominent position in Chinese pharmacopeia, historically employed in managing diabetes, hyperlipidemia, atherosclerosis, and diverse malignancies. Cycloastragenol (CAG), a key bioactive constituent of *Astragalus membranaceus*, exhibits anti‐senescence, antioxidant, and anti‐inflammatory activities [30, 31, 32, 33], but its potential to ameliorate PFAS‐induced cardiac senescence and the underlying molecular mechanism are completely unknown.

To address these critical knowledge gaps, we hypothesized that PFOA/HFPO‐TA exposure suppresses CDK4 expression to destabilize MERCs, impair PINK1/Parkin‐mediated mitophagy, trigger mtDNA leakage and cGAS‐STING activation, and ultimately induce cardiac senescence, with HFPO‐TA exerting stronger toxic effects. Here, we combined in vivo and in vitro models to elucidate the regulatory role of the CDK4/MERCs/PINK1 axis in PFOA/HFPO‐TA‐induced cardiac senescence, and to verify whether CAG ameliorates this toxic process by restoring CDK4 expression and stabilizing MERCs. This work aims to identify novel molecular targets for PFAS‐associated cardiovascular toxicity assessment and mitigation and to provide experimental evidence for natural product‐based interventions against environmental pollutant‐induced cardiac senescence, filling key gaps in the non‐hepatic toxicology research of PFAS alternatives.

## 2 Results

### 2.1 PFOA/HFPO‐TA Exposure Induces Cardiac Structural and Functional Damage With HFPO‐TA Exhibiting Higher Potency

To explore the effects of PFOA and HFPO‐TA on the heart, we established an in vivo mouse model (Figure 1A). Histological staining showed that the cardiac structure in the Con group was intact, and the cardiomyocytes were arranged regularly. In contrast, the PFOA and HFPO‐TA exposure groups exhibited disordered cardiac tissue structure, loosely arranged cardiomyocytes, and inflammatory cell infiltration (Figure 1B). Accordingly, PFOA and HFPO‐TA exposure significantly increased the cardiac pathological score compared with the Con group (Figure 1C). Cardiac Troponin T (cTNT) is a protein specifically expressed in cardiomyocytes that serves as a sensitive indicator of minor cardiomyocyte damage. To assess the effects of PFOA and HFPO‐TA on the cardiac structure, we detected the expression of cTNT.

**FIGURE 1:**
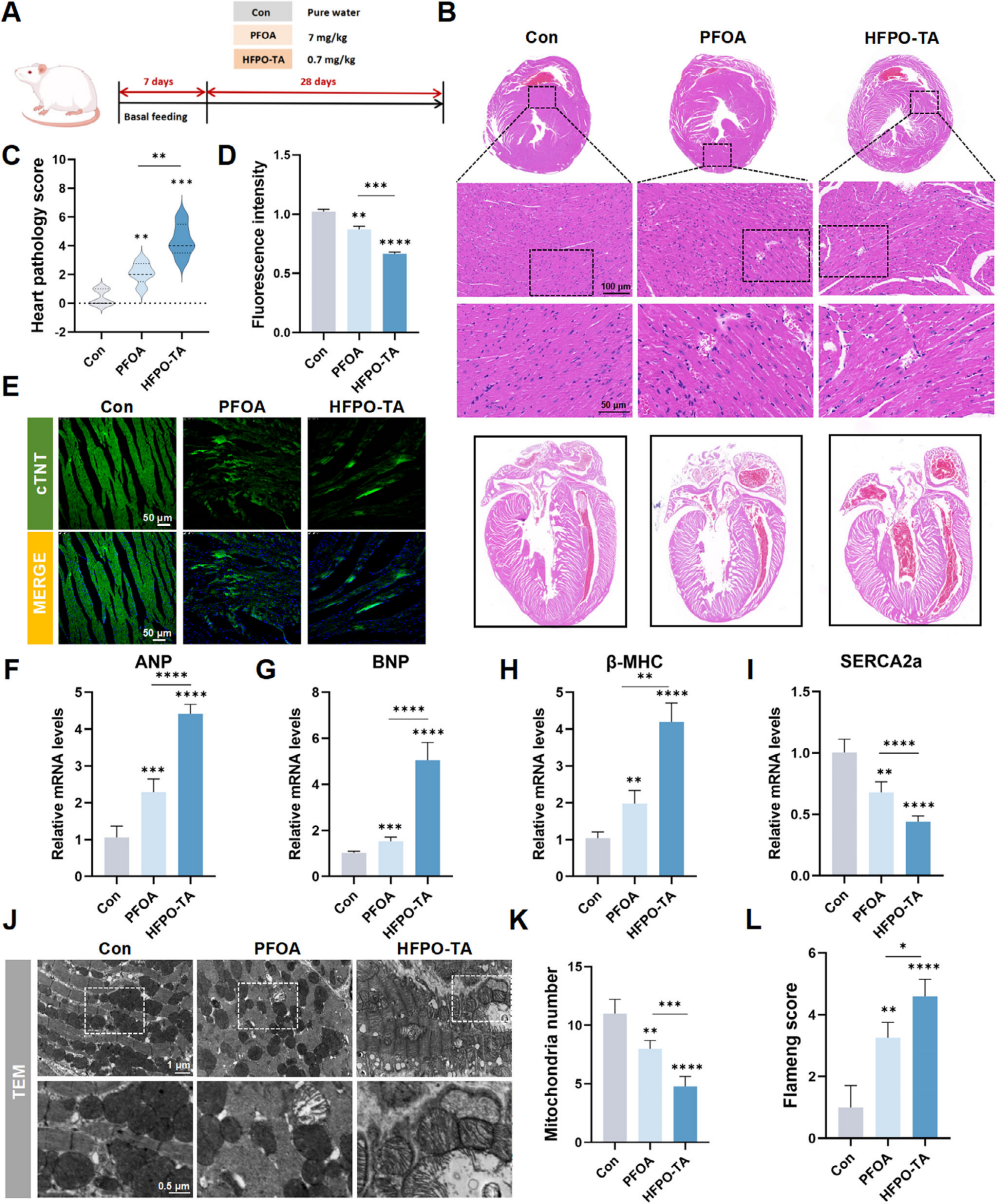
PFOA/HFPO‐TA exposure induces cardiac structural and functional damage. (A) The mice were treated with PFOA/HFPO‐TA. (B) Histopathology with H&E staining (×200 and ×400). (C) Heart pathology score. (D‐E) Representative IF images of cTNT and fluorescence intensity. (F‐I) qRT‑PCR analysis in heart tissues. (J) TEM analysis. (K) Number of mitochondria. (L) Flameng score. Data are presented as the mean ± SD, *n*  =  3. ^*^ *p* < 0.05, ^**^ *p* < 0.01, ^***^ *p* < 0.001, ^****^ *p* < 0.0001.

Our results showed that cTNT levels were significantly reduced in both exposure groups, with HFPO‐TA causing a more severe reduction than PFOA (Figure 1D,E). To further evaluate cardiac function, we measured the mRNA expression of heart failure markers. Both PFOA and HFPO‐TA significantly upregulated *ANP, BNP, and β‐MHC*, while downregulating *SERCA2a*, indicating cardiac stress, contractile dysfunction, and impaired diastolic function, with HFPO‐TA showing stronger effects (Figure 1F–I). Concomitantly, subcellular mitochondrial structural abnormalities were observed. Transmission electron microscopy (TEM) revealed the accumulation of damaged mitochondria. In the Con group, the mitochondrial structure was normal, with intact membranes and cristae. However, in the PFOA and HFPO‐TA exposure groups, the outer mitochondrial membranes were ruptured, the cristae were damaged, and mitochondrial vacuoles were observed, accompanied by an increase in Flameng score and a decrease in the number of mitochondria (Figure 1J–L). Collectively, these results demonstrate that exposure to PFOA or HFPO‐TA causes severe structural and functional damage to the heart.

### 2.2 PFOA/HFPO‐TA Exposure Induces Mitochondrial Dysfunction, Impaired Mitophagy, and mtDNA Accumulation

The maintenance of cardiac homeostasis highly depends on mitochondria, and mitochondrial quality control mechanisms are crucial [34]. Detection of mitochondrial ROS levels in cardiac tissues showed that PFOA and HFPO‐TA exposure significantly increased mitochondrial ROS levels (Figure 2A). Mitophagy can eliminate damaged mitochondria in cells. The autophagic flux marker p62 was significantly upregulated, whereas the pro‐mitophagic and antioxidant proteins Beclin 1, Sirt3, and SOD2 were markedly downregulated in the PFOA and HFPO‐TA groups. Notably, the LC3‐II/I ratio was decreased, and Parkin and PINK1 expression levels were also reduced, indicating impaired autophagic activity (Figure 2B,C). PINK1/Parkin‐dependent mitophagy is the main pathway for maintaining mitochondrial homeostasis. We therefore investigated the effect of PFOA and HFPO‐TA exposure on PINK1/Parkin‐mediated mitophagy. Consistently, immunofluorescence staining of heart tissues confirmed a significant decrease in PINK1 and Parkin fluorescence intensity upon PFOA and HFPO‐TA exposure (Figure 2D). Impaired mitophagy leads to the inability to clear damaged mitochondria in a timely manner, which in turn triggers the abnormal release and accumulation of mtDNA (Figure 2E) [35].

**FIGURE 2:**
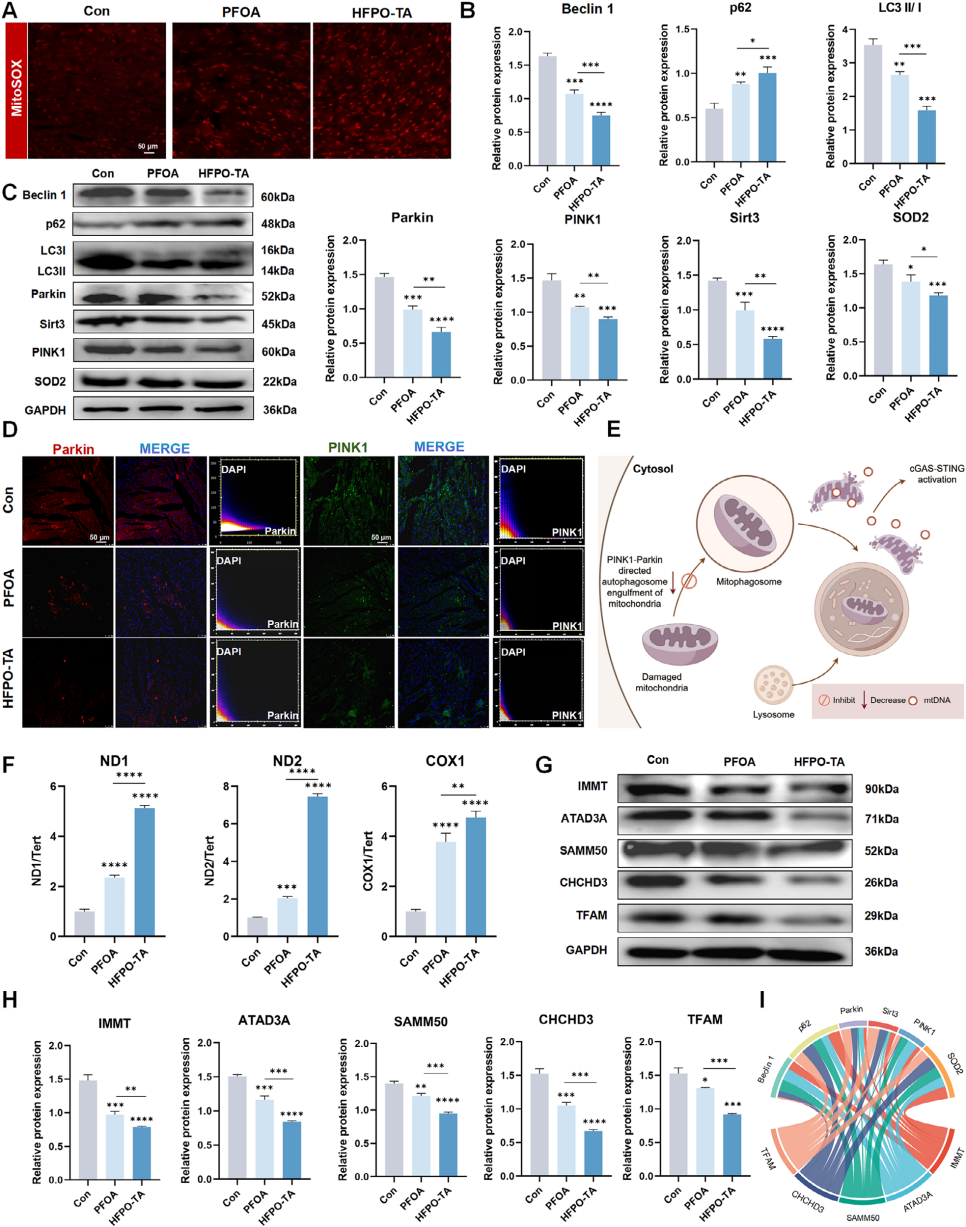
PFOA/HFPO‐TA exposure induces mitophagy impairment. (A) Mitochondrial ROS level. (B‐C) Western blotting images and quantitative results of proteins. (D) Representative IF images of Parkin and PINK1. (E) Schematic diagram depicting the accumulation of mtDNA and the activation of the cGAS‐STING pathway induced by mitophagy impairment. (F) qPCR analysis of cytosolic mtDNA levels. (G‐H) Protein levels of IMMT, ATAD3A, SAMM50, CHCHD3 and TFAM. (I) Chord diagrams. Data are presented as the mean ± SD, *n*  =  3. ^*^ *p* < 0.05, ^**^ *p* < 0.01, ^***^ *p* < 0.001, ^****^ *p* < 0.0001.

To directly assess whether mitophagy impairment leads to mtDNA leakage, we quantified cytosolic mtDNA levels by qPCR. The purity of the cytosolic fraction was validated by Western blotting, which showed robust GAPDH signal and undetectable Lamin B, confirming no nuclear contamination (Figure S1). Both PFOA and HFPO‐TA significantly increased the cytoplasmic copy numbers of the mitochondrial genes *ND1, ND2, and COX1*, with HFPO‐TA inducing more pronounced elevations (Figure 2F). Moreover, as shown in Figure 2G,H, PFOA treatment reduced the levels of TFAM as well as multiple MICOS constituents and associated proteins, including SAMM50, IMMT, CHCHD3 and ATAD3A. This reduction was more significant upon exposure to HFPO‐TA. The accumulation of mtDNA was closely related to PINK1/Parkin pathway‐mediated mitophagy (Figure 2I). In conclusion, these results indicate that PFOA and HFPO‐TA induce mitochondrial dysfunction, impaired PINK1/Parkin‐mediated mitophagy, and subsequent mtDNA accumulation, with HFPO‐TA exerting more potent effects.

### 2.3 PFOA/HFPO‐TA Exposure Activates the cGAS‐STING Pathway to Drive Cardiac Senescence

Mitochondrial DNA (mtDNA) that leaks into the cytoplasm, as abnormal double‐stranded DNA, is recognized by cGAS, leading to activation of cGAS signaling. Western blotting results in Figure 3A,B showed that compared with the Con group, the levels of cGAS, STING, PERK, and eIF2α were significantly increased in the PFOA/HFPO‐TA groups, indicating activation of the cGAS‐STING pathway upon PFOA/HFPO‐TA exposure. These results suggest that PFOA/HFPO‐TA activates the cGAS‐STING‐PERK‐eIF2α axis through mtDNA accumulation, a known driver of cellular senescence [23]. SA‐β‐gal staining revealed that the positively stained area was increased after PFOA/HFPO‐TA exposure compared with the Con group (Figure 3C). DNA damage is associated with cellular senescence. The enhanced expression of p53, p21, p16, and the release of the SASP are hallmarks of cellular senescence. As quantified in Figure 3D–F, exposure to PFOA or HFPO‐TA upregulated the protein levels of p53, p‐p53, p21, and p16, while downregulating CDK4. Additionally, the mRNA levels of the SASP factors *IL‐6*, *IL‐1β*, *TNF‐α*, and *CCL2* were significantly elevated upon PFOA/HFPO‐TA exposure (Figure 3G). Consistently, immunofluorescence staining demonstrated a substantially larger p21/p16‑positive area in the PFOA/HFPO‐TA‑treated groups (Figure 3H). To establish causal evidence, we performed cGAS knockdown and STING pharmacological inhibition in HL‐1 cardiomyocytes. To assess cytotoxicity, HL‐1 cells were incubated with escalating concentrations of PFOA or HFPO‐TA (Figure S2).

**FIGURE 3:**
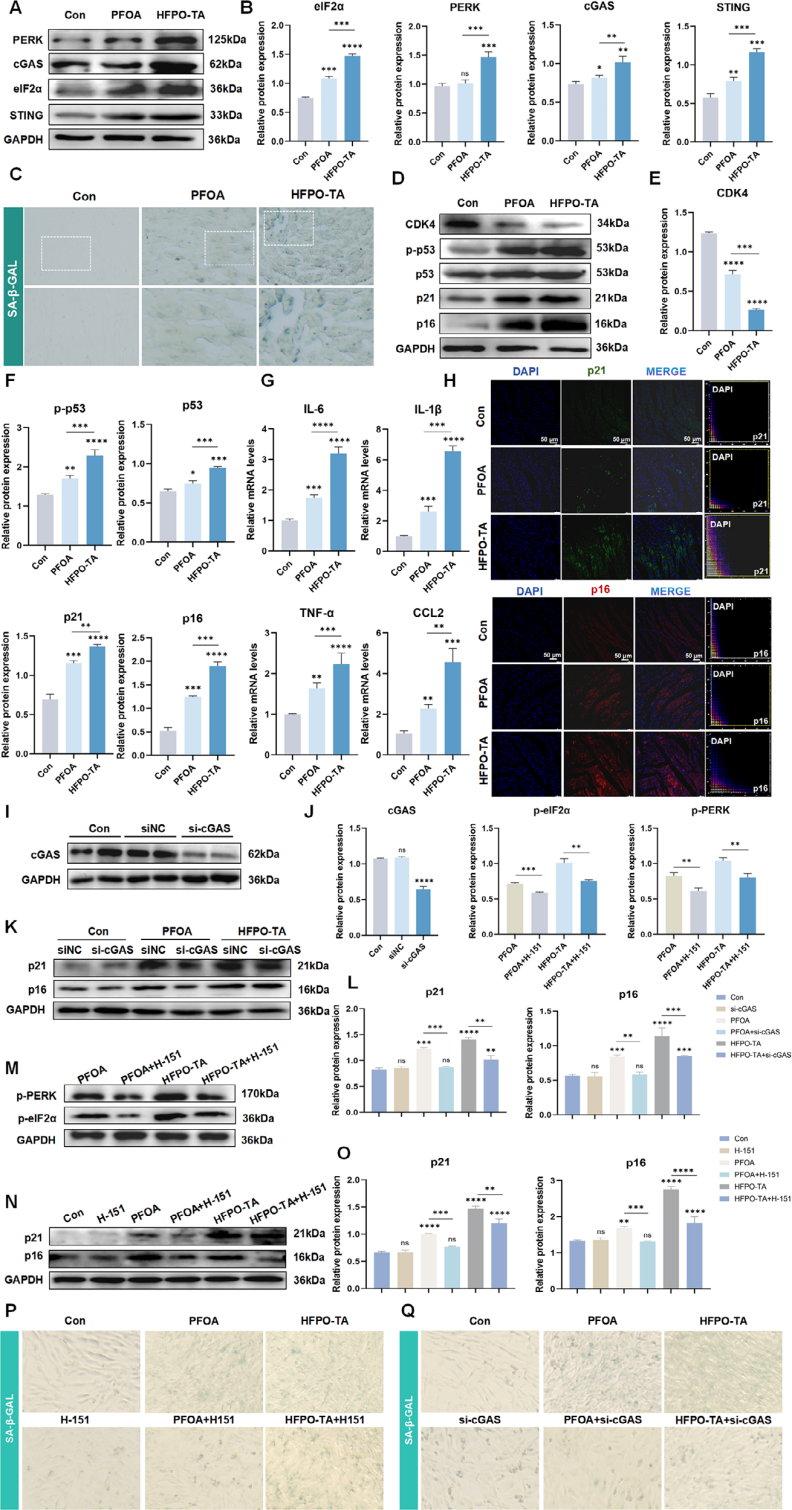
PFOA/HFPO‐TA exposure activates the cGAS‐STING pathway and induces cardiac senescence. (A,B) Protein levels of PERK, cGAS, eIF2α and STING. (C) SA‐β‐gal staining images of hearts from different groups. (D–F) Western blotting images and quantitative results of proteins. (G) qRT‐PCR analysis of SASP factors in heart tissues. (H) Representative IF images and fluorescence intensity of p21 and p16 proteins. (I) Validation of cGAS knockdown efficiency in HL‐1 cells. (J) Quantitative results of cGAS, p‐PERK, and p‐eIF2α. (K,L) Protein levels of p21 and p16 in cGAS knockdown experiments. (M) Western blot analysis of HL‐1 cells treated with H‐151. (N‐O) Protein levels of p21 and p16 in HL‐1 cells treated with H‐151. (P,Q) SA‐β‐gal staining of HL‑1 cells from cGAS knockdown and STING inhibitor experiments. Data are presented as the mean ± SD, *n*  =  3. ^*^ *p* < 0.05, ^**^ *p* < 0.01, ^***^ *p* < 0.001, ^****^ *p* < 0.0001.

Based on our CCK‐8 viability results and previous studies [36, 37], we selected 100 µM PFOA and 50 µM HFPO‐TA for subsequent experiments. cGAS knockdown was validated by Western blotting, showing a marked reduction in cGAS protein compared with siNC controls (Figure 3I,J). Functionally, cGAS knockdown significantly attenuated PFOA/HFPO‐TA‐induced upregulation of p21 and p16 (Figure 3K,L). Treatment with the STING inhibitor H‐151 at 1 µM effectively reduced the p‐PERK and p‐eIF2α levels induced by PFOA or HFPO‐TA (Figure 3M,J). Similarly, STING inhibition with H‐151 also rescued the PFOA/HFPO‐TA‐induced increase in p21 and p16 (Figure 3N,O). SA‐β‐gal staining further confirmed that both cGAS knockdown and STING inhibition markedly alleviated the senescent phenotype (Figure 3P,Q). Collectively, these results demonstrate that PFOA and HFPO‐TA drive cardiac senescence through the cGAS‐STING‐PERK‐eIF2α axis, with HFPO‐TA consistently exhibiting stronger effects than PFOA.

### 2.4 PFOA/HFPO‐TA Exposure Causes MERCs Disruption Associated With Cardiac Senescence

MERCs are the sites where autophagy is initiated, and autophagosomes are formed [38]. MERCs influence cellular senescence by regulating processes such as mitochondrial cristae structure, mtDNA stability, and mitophagy. To illustrate the structural and functional changes of MERCs induced by PFOA/HFPO‐TA, we constructed a schematic diagram (Figure 4A), which depicts the disruption of MERCs tethers upon PFOA/HFPO‐TA exposure. MERCs mainly consist of several tethers: MFN1/MFN2 heterodimers, MFN2 homodimers, VDAC‐GRP75‐IP3R, and FUNDC1‐IP3R [39]. Western blotting results in Figure 4B,C showed that exposure to PFOA and HFPO‐TA downregulated the expression of MFN2, FUNDC1, and IP3R1. To directly assess MERCs ultrastructure, we performed TEM analysis. As shown in Figure 4D, TEM revealed close apposition between mitochondrial and endoplasmic reticulum (ER) membranes in control hearts, whereas PFOA or HFPO‐TA exposure caused significant separation and reduced contact sites. Quantitative analysis of TEM images (Figure 4E) demonstrated that PFOA and HFPO‐TA significantly decreased MERCs coverage per unit area, increased the average distance between mitochondria and ER, and decreased MERCs length. These results provide direct structural evidence that PFOA and HFPO‐TA disrupt MERCs integrity. Consistent with these ultrastructural changes, fluorescence co‐localization imaging using TOM20 (mitochondrial marker) and Calnexin (ER marker) showed reduced co‐localization of mitochondria and ER in the PFOA and HFPO‐TA groups (Figure 4F), confirming MERCs disruption. Correlation analysis revealed that MERCs were significantly correlated with genes involved in mitophagy and senescence (Figure 4G). Additionally, PPI network analysis further identified MERCs‐related proteins as core hubs connecting autophagic signaling and senescence pathways (Figure 4H), reinforcing the functional link between MERCs disruption and cardiac senescence. In summary, these data indicate that exposure to PFOA/HFPO‐TA leads to MERCs dysregulation, which contributes to cardiac senescence caused by impaired mitophagy.

**FIGURE 4:**
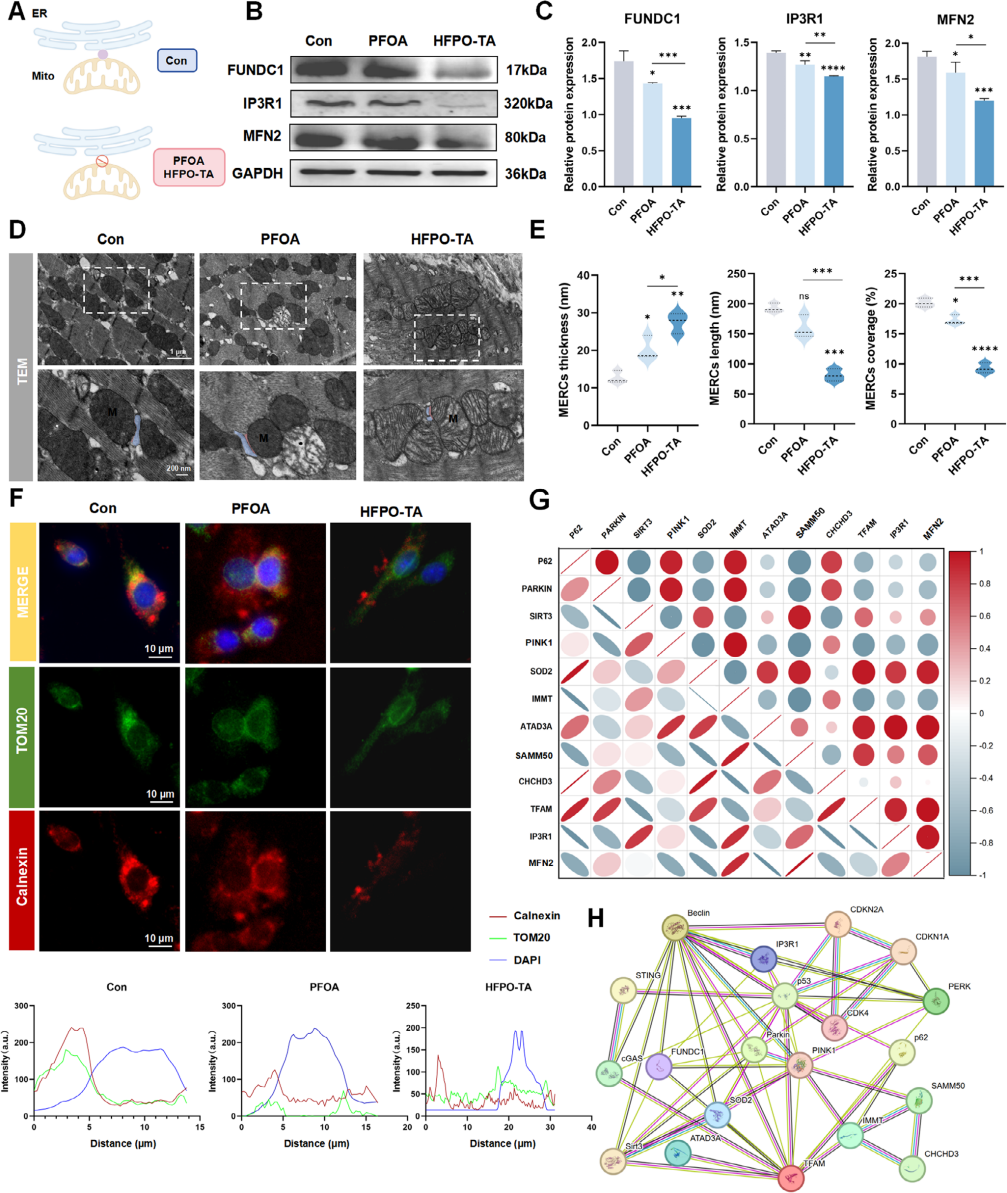
PFOA/HFPO‐TA exposure induces MERCs disruption. (A) Schematic diagram illustrating MERCs disruption caused by PFOA/HFPO‐TA exposure. (B,C) Protein levels of FUNDC1, IP3R1 and MFN2. (D) Representative TEM images from hearts used to analyze MERCs. The MERCs were shown in red and the ER was shown in blue. (E) MERCs thickness, length and coverage in hearts. (F) Representative IF images and fluorescence intensity of MERCs, TOM20 indicated mitochondria and Calnexin indicated ER. (G) Correlation analysis. (H) PPI network. Data are presented as the mean ± SD, *n*  =  3. ^*^ *p* < 0.05, ^**^ *p* < 0.01, ^***^ *p* < 0.001, ^****^ *p* < 0.0001.

### 2.5 PFOA/HFPO‐TA Exposure Impairs the Mitochondrial PINK1 Localization and Autophagic Flux in HL‐1 Cells

To further evaluate the potential molecular mechanisms of PFOA and HFPO‐TA in cardiomyocytes, an in vitro model was established. Mirroring our in vivo observations, cell morphology analysis revealed that PFOA and HFPO‐TA caused obvious damage to cardiomyocytes (Figure 5A). Moreover, both compounds significantly elevated intracellular ROS levels and reduced mitochondrial membrane potential (MMP) (Figure 5B). Normally, MERCs provide spatial support for the localization of PINK1, and disruption of MERCs can impede this process, leading to the accumulation of damaged mitochondria. Fluorescence co‐localization imaging using Mito‐DsRed and PINK1 antibody showed that the mitochondrial localization of PINK1 was significantly impaired in the PFOA and HFPO‐TA groups, as evidenced by reduced overlap and fluorescence intensity, with HFPO‐TA causing more severe disruption (Figure 5C). To assess autophagic flux, we treated HL‐1 cells with bafilomycin A1 (Baf A1), an inhibitor of autophagosome‐lysosome fusion. Western blotting showed that PFOA or HFPO‐TA alone increased p62 and decreased LC3‑II/I ratio, indicating impaired autophagic flux. Co‐treatment with Baf A1 further increased the LC3‑II/I ratio and p62 accumulation, indicating that PFOA/HFPO‐TA suppressed downstream autophagosome clearance rather than blocking autophagosome formation (Figure 5D). Quantification of these changes is presented in Figure 5F. We next examined senescence markers. Exposure to PFOA or HFPO‑TA significantly upregulated p21 and p16, and this upregulation was further exacerbated by Baf A1 co‑treatment (Figure 5E,G). SA‐β‐gal staining confirmed that PFOA/HFPO‑TA exposure increased the percentage of senescent cells, which was further increased by Baf A1 (Figure 5H). A schematic diagram summarizing the mechanism of impaired PINK1 localization and autophagic flux is presented in Figure 5I. Collectively, these results demonstrate that PFOA and HFPO‑TA impair PINK1 mitochondrial recruitment and autophagic flux, and that blockade of autophagy exacerbates senescence, supporting a causal role for mitophagy defect in PFOA/HFPO‑TA‑induced cardiac senescence.

**FIGURE 5:**
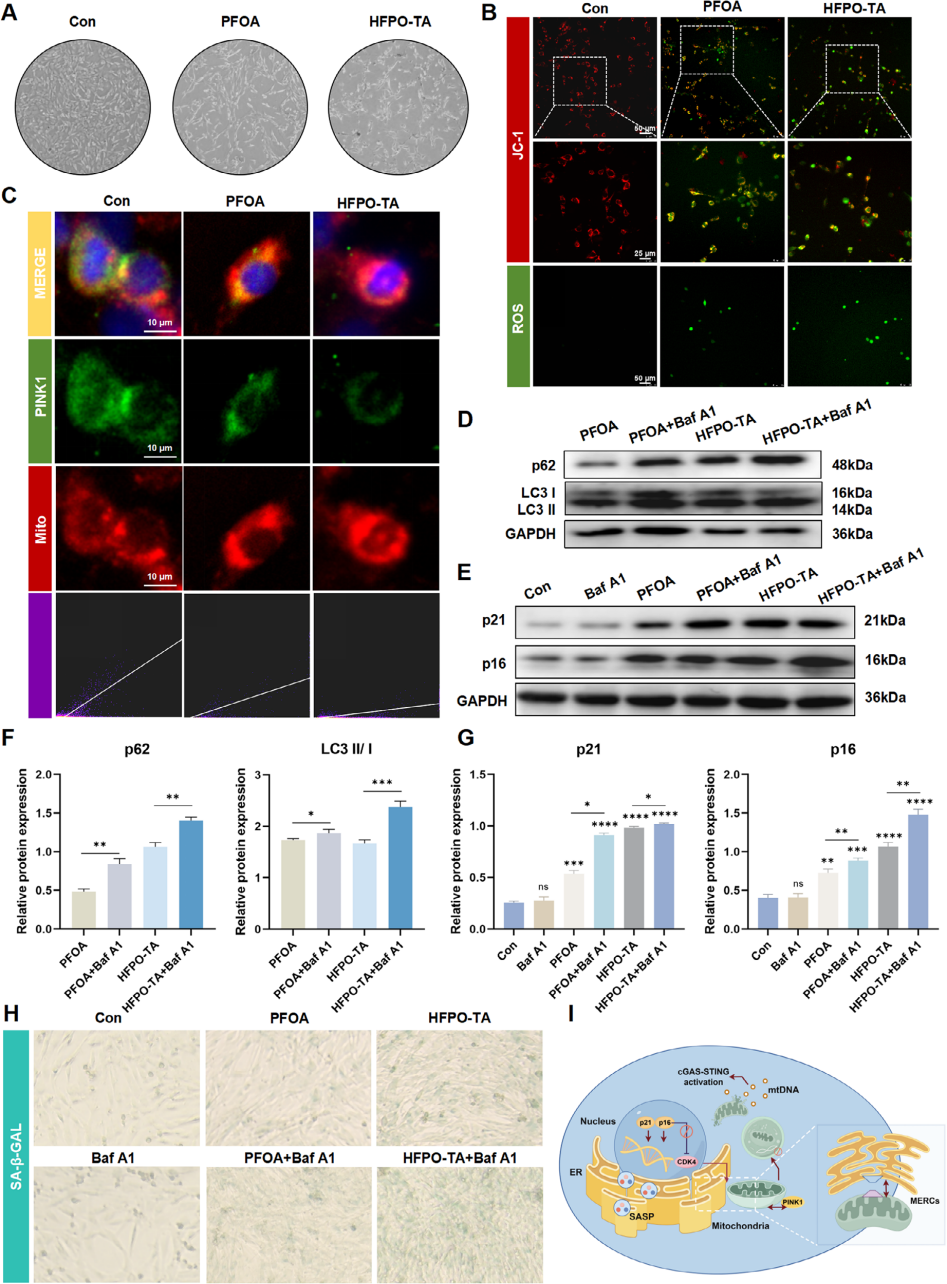
PFOA/HFPO‐TA impairs PINK1 mitochondrial localization and autophagic flux in HL‐1 cells. (A) Microscopic observation was made after culture. (B) MMP and ROS levels. (C) Representative IF images of PINK1. HL‐1 cells were transfected with Mito‐DsRed. (D) Western blot analysis of p62 and LC3‐II/I protein expression under Baf A1 treatment. (E) Western blot analysis of p21 and p16 protein expression under Baf A1 treatment. (F‐G) Quantitative results of proteins. (H) SA‐β‐gal staining under Baf A1 treatment. (I) Schematic diagram illustrating impaired PINK1 mitochondrial localization and mitophagic flux caused by MERCs disruption. Data are presented as the mean ± SD, n  =  3. ^*^ *p* < 0.05, ^**^ *p* < 0.01, ^***^ *p* < 0.001, ^****^ *p* < 0.0001.

### 2.6 CDK4 Acts Upstream of MERCs and PINK1 to Regulate PFOA/HFPO‐TA‐Induced Senescence in HL‐1 Cells

In senescent cells, p16 binds to CDK4 and inhibits its activity, resulting in impaired CDK4 function and subsequent cell cycle arrest. Inactivation of CDK4 affects MERCs [27]. To determine whether CDK4 downregulation precedes and thereby drives MERCs disruption and senescence upon PFOA and HFPO‐TA exposure, we selected time points of 2 h, 6 h, and 12 h to investigate the changes in CDK4 and MERCs during the exposure. As shown in Figure 6A, the SA‐β‐gal‐positive area increased progressively with prolonged exposure, with HFPO‐TA showing a slightly larger effect than PFOA. Fluorescence imaging revealed that CDK4 expression decreased in a time‐dependent manner (Figure 6B,C). Concurrently, MERCs integrity gradually declined (Figure 6C,D). Notably, CDK4 downregulation occurred prior to both MERCs reduction and the increase in SA‑β‑gal‑positive area, suggesting that CDK4 inhibition is an upstream event in PFOA/HFPO‑TA‑induced MERCs dysregulation and cardiomyocyte senescence. Molecular docking analysis further revealed the direct binding of PFOA and HFPO‐TA to CDK4, with binding free energies of ‐8.2 kcal/mol and ‐10.5 kcal/mol, respectively (Figure 6E,F), indicating a strong binding affinity, with HFPO‐TA showing a theoretically tighter interaction with CDK4 than PFOA.

**FIGURE 6:**
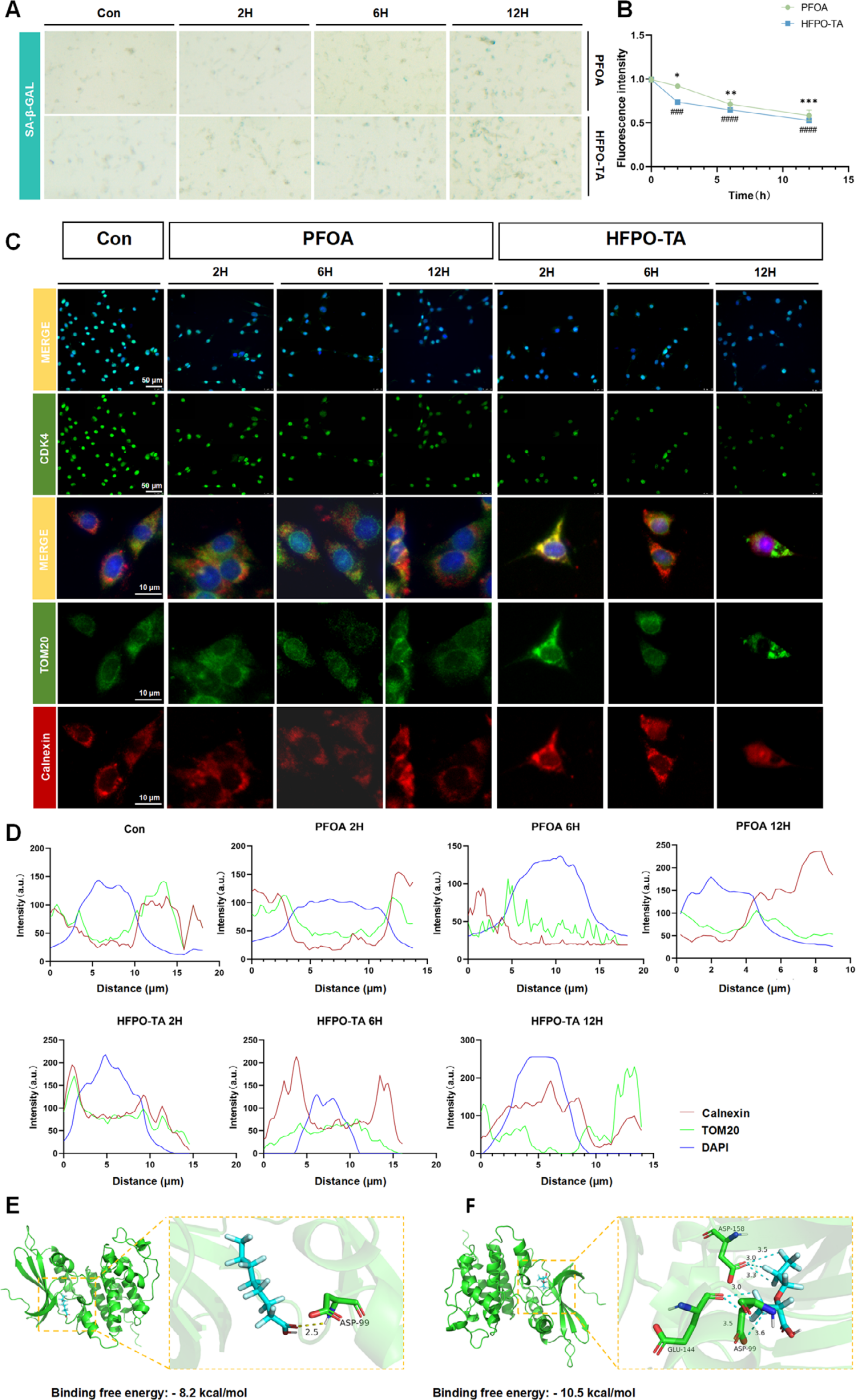
Effect of CDK4 on PFOA/HFPO‐TA‐induced MERCs disruption and senescence in HL‐1 cells. (A) SA‐β‐gal staining images. (B) Fluorescence intensity of CDK4 protein. (C‐D) Representative IF images of CDK4 protein and MERCs, TOM20 indicated mitochondria and Calnexin indicated ER. (E) Molecular docking simulation of PFOA with CDK4. (F) Molecular docking simulation of HFPO‐TA with CDK4. Data are presented as the mean ± SD, *n*  =  3. ^*^ *p* < 0.05, ^**^ *p* < 0.01, ^***^ *p* < 0.001, ^****^ *p* < 0.0001, ^###^ *p* < 0.001, ^####^ *p* < 0.0001.

To directly test whether restoring CDK4 expression is sufficient to counteract the observed effects, we performed CDK4 overexpression experiments (Figure 7A). Western blotting confirmed that CDK4 protein levels were markedly increased in CDK4‐overexpressing (OE‐CDK4) cells compared to empty vector (Vector) controls (Figure 7B).

**FIGURE 7:**
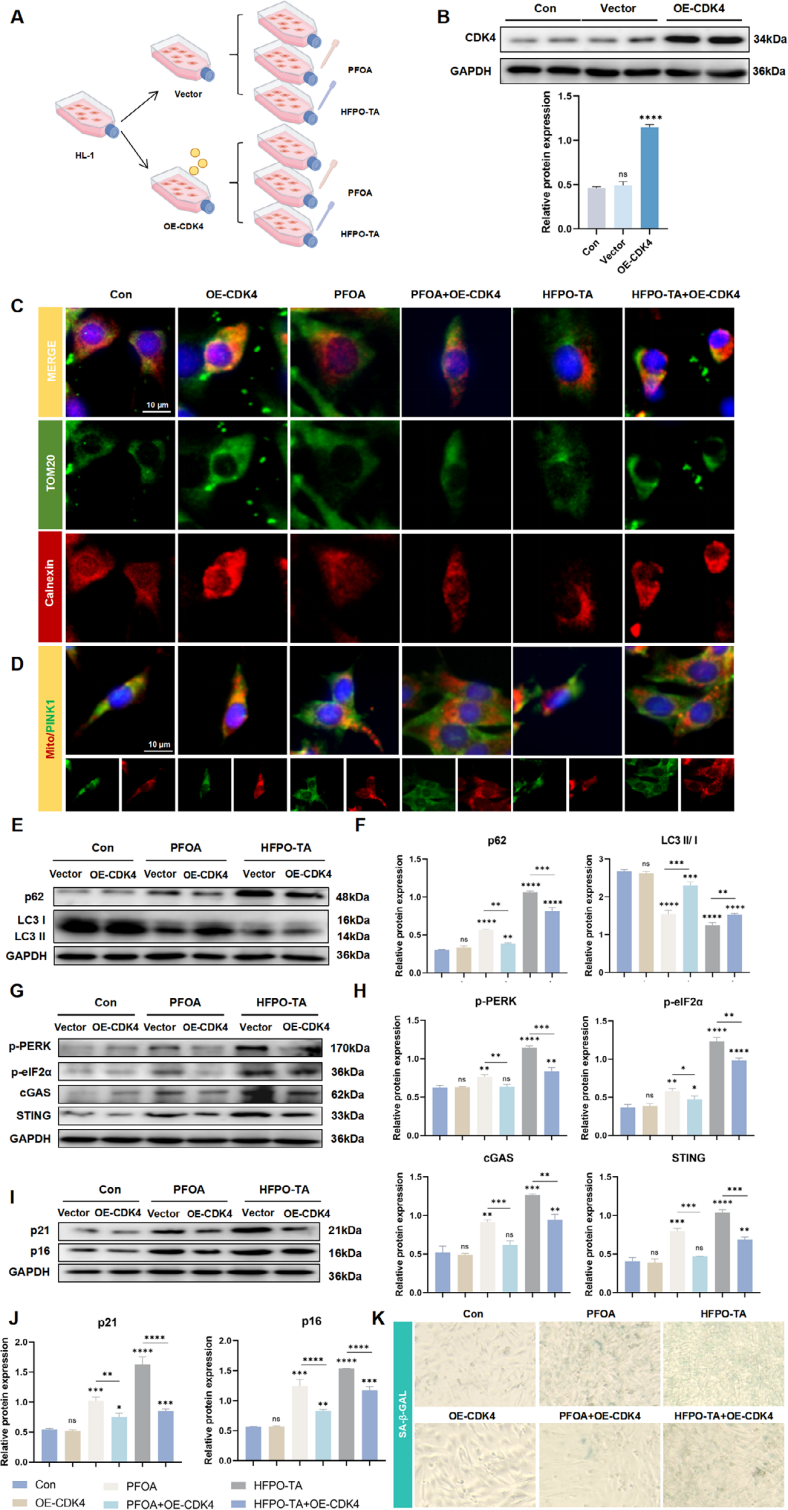
CDK4 overexpression rescues PFOA/HFPO‐TA‐induced senescence and cGAS‐STING activation. (A) Schematic diagram of CDK4 overexpression rescue experiment. (B) Western blot validation of CDK4 overexpression in HL‐1 cells. (C) Representative IF images of MERCs, TOM20 indicated mitochondria and Calnexin indicated ER. (D) Representative IF images of PINK1. HL‐1 cells were transfected with Mito‐DsRed. (E,F) Protein levels of p62 and LC3‐II/I. (G‐H) Protein levels of p‐PERK, cGAS, p‐eIF2α and STING. (I,J) Protein levels of p21 and p16. (K) SA‐β‐gal staining images. Data are presented as the mean ± SD, *n*  =  3. ^*^ *p* < 0.05, ^**^ *p* < 0.01, ^***^ *p* < 0.001, ^****^ *p* < 0.0001.

As shown in Figure 7C, PFOA/HFPO‐TA treatment reduced TOM20‐Calnexin co‐localization, indicating MERCs disruption, whereas CDK4 overexpression significantly restored MERCs integrity. Consistently, CDK4 overexpression rescued the impaired mitochondrial localization of PINK1 (Figure 7D) and normalized autophagic flux, as evidenced by reversal of the increased p62 and decreased LC3‑II/I ratio (Figure 7E,F). Moreover, CDK4 overexpression suppressed the PFOA/HFPO‐TA‑induced activation of the cGAS‐STING pathway (Figure 7G,H). Consequently, the PFOA/HFPO‐TA‐induced increase in p21 and p16 was markedly attenuated by CDK4 overexpression (Figure 7I,J). Consistently, CDK4 overexpression also reduced the mRNA levels of SASP factors, including *IL‑6*, *IL‑1β*, *TNF‑α*, and *CCL2* (Figure S3). SA‑β‑gal staining further confirmed alleviation of the senescent phenotype (Figure 7K). Collectively, these results demonstrate that CDK4 is a critical upstream regulator of the CDK4/MERCs/PINK1 axis, and that restoring CDK4 expression is sufficient to rescue PFOA/HFPO‐TA‐induced mitophagy defect, cGAS‐STING activation, and cardiac senescence.

### 2.7 CAG Binds to CDK4 and Restores MERCs Stability to Mitigate PFOA/HFPO‐TA‐Induced Senescence in HL‐1 Cells

CAG has been reported to possess anti‐senescence pharmacological activity [40]. To investigate whether CAG ameliorates cardiac senescence induced by PFOA/HFPO‐TA, we established an in vitro model with combined treatment of CAG and PFOA/HFPO‐TA. Exposure of HL‐1 cells to escalating concentrations of CAG revealed that 60 µM CAG exerted no effect on cell viability. Based on existing studies [41, 42, 43], we selected 10 µM CAG for subsequent experiments (Figure 8A). Molecular docking analysis predicted direct binding between CAG and CDK4, forming a stable complex (Figure 8B). Both Western blotting and fluorescence imaging demonstrated that CAG treatment alleviated the inhibition of CDK4 caused by PFOA/HFPO‐TA (Figure 8C–E). CAG significantly restored MERCs integrity, as evidenced by increased co‐localization of TOM20 and Calnexin in PFOA/HFPO‐TA‐exposed cells (Figure 8F). We also found that CAG could restore the decreased migration ability of HL‐1 cells induced by PFOA and HFPO‐TA (Figure S4A). The cell morphological results showed that CAG could restore the cell damage induced by PFOA and HFPO‐TA (Figure S4B). CAG treatment could also reduce the increase in ROS levels induced by PFOA and HFPO‐TA (Figure S4C).

**FIGURE 8:**
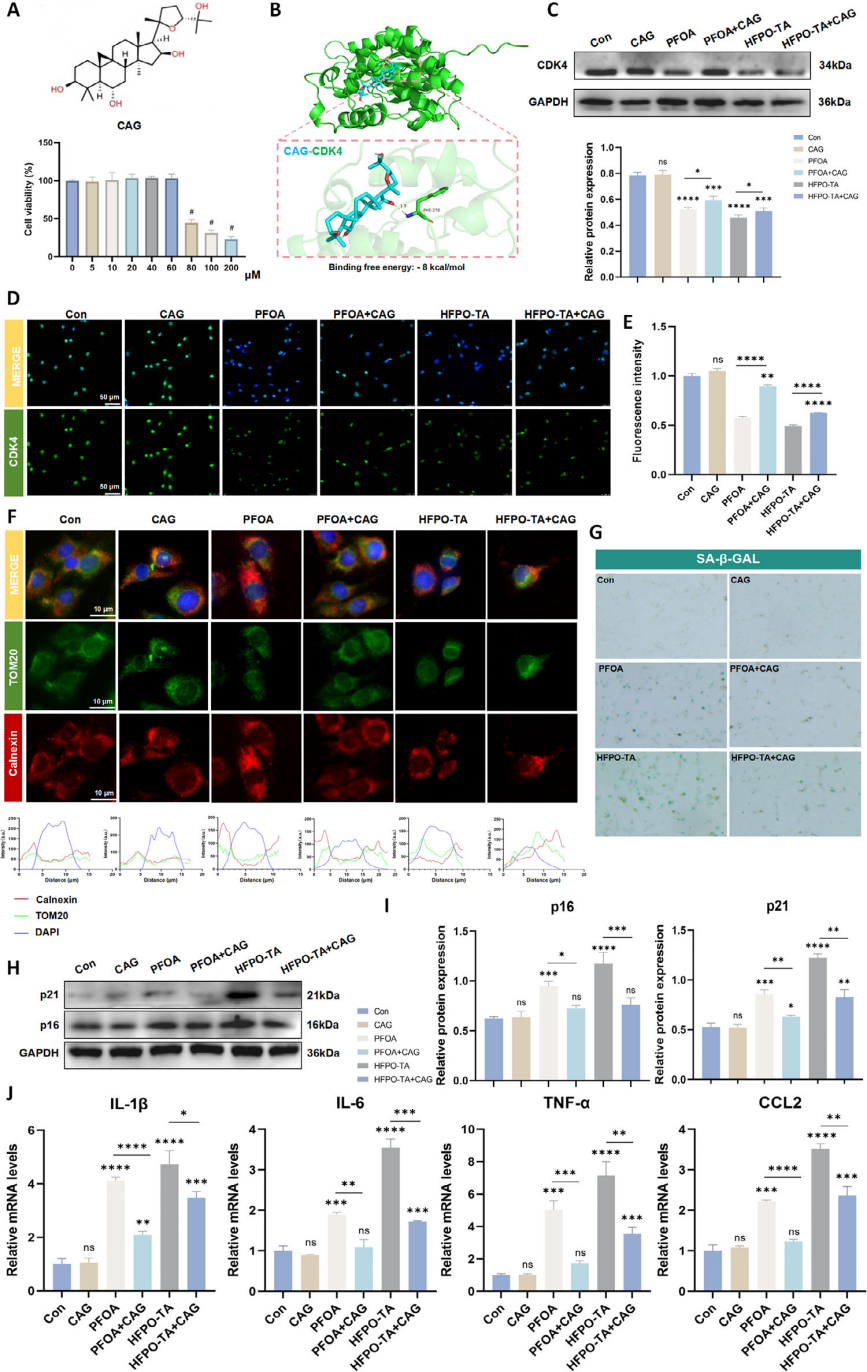
CAG mitigates PFOA/HFPO‐TA‐induced CDK4 and MERCs disruption in HL‐1 cells. (A) Chemical structure of CAG and CCK‐8 assay. (B) Molecular docking simulation of CAG with CDK4. (C) Protein level of CDK4. (D‐E) Representative IF images and fluorescence intensity of CDK4 protein. (F) Representative IF images of MERCs, TOM20 indicated mitochondria and Calnexin indicated ER. (G) SA‐β‐gal staining images. (H‐I) Protein levels of p21 and p16. (J) qRT‐PCR analysis of SASP factors. Data are presented as the mean ± SD, *n*  =  3. ^*^ *p* < 0.05, ^**^ *p* < 0.01, ^***^ *p* < 0.001, ^****^ *p* < 0.0001.

Consistent with these protective effects, CAG markedly reduced the PFOA/HFPO‑TA‑induced increase in SA‑β‑gal‑positive area (Figure 8G), downregulated the senescence markers p21 and p16 (Figure 8H,I), and suppressed the mRNA levels of SASP factors *IL‑6*, *IL‑1β*, *TNF‑α*, and *CCL2* (Figure 8J). Collectively, these results demonstrate that CAG can restore CDK4 expression and MERCs integrity, further alleviating PFOA/HFPO‐TA‐induced myocardial cell damage and senescence (Figure 9).

**FIGURE 9:**
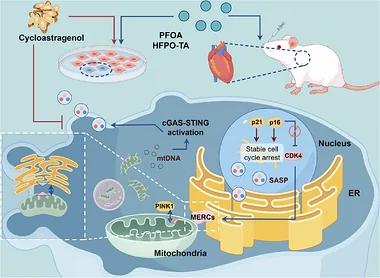
The working model illustrating that PFOA/HFPO‐TA exposure triggers cardiac senescence via suppressing the CDK4/MERCs/PINK1 axis to impair mitophagy and promote cGAS‐STING signaling activation, while CAG reverses these pathological processes and ultimately mitigates PFOA/HFPO‐TA‐induced cardiac senescence.

## 3 Discussion

The global production and environmental release of PFAS continue to rise, raising concerns for public health. Although numerous studies have documented the multi‑organ toxicity of PFOA and its replacement HFPO‑TA, the mechanisms responsible for their cardiotoxicity remain incompletely understood. The heart is highly susceptible to senescence due to its high energy demands. Cardiac senescence contributes to functional decline, the pathogenesis of heart diseases, and diminished life quality. Emerging evidence indicates exposure to environmentally harmful substances may also accelerate organ senescence and induce diseases [44]. The present study identifies a novel toxicological axis, CDK4/MERCs/PINK1, as the central mediator of PFOA/HFPO‐TA‐induced cardiac senescence, and further verifies that CAG exerts a protective effect by targeting this axis. Collectively, our findings elucidate the mechanistic basis linking PFAS exposure to cardiac senescence and provide essential experimental evidence for assessing the health risks of PFAS alternatives and advancing natural product‐based therapeutic strategies against pollutant‐induced organ damage.

A key finding of this study is that HFPO‐TA exerts more potent cardiac toxic effects than PFOA at the tested dose, which aligns with previous observations of higher hepatic and serum accumulation of HFPO‐TA in mammalian models [15, 16, 17, 45]. This enhanced toxicity is not a mere phenotypic change but is driven by a more severe disruption of the entire CDK4/MERCs/PINK1 regulatory axis. HFPO‐TA induces a more significant downregulation of CDK4, leading to greater destabilization of MERCs tether proteins, more severe impairment of PINK1 mitochondrial recruitment, and a stronger inhibition of PINK1/Parkin‐mediated mitophagy. The exacerbated mitophagy defect further results in more profound mitochondrial structural damage and a more significant reduction in cristae‐associated proteins, ultimately triggering excessive mtDNA leakage into the cytosol. This elevated mtDNA leakage activates the cGAS‐STING pathway more robustly, leading to a more pronounced upregulation of senescence markers and a higher SA‐β‐gal positive rate, forming a vicious cycle of mitochondrial dysfunction and cardiac senescence. Collectively, these data confirm that the differential potency of PFOA and HFPO‐TA in inducing cardiac senescence is rooted in their differential ability to suppress the CDK4/MERCs/PINK1 axis, with HFPO‐TA causing a more complete disruption of this regulatory network.

Mitochondria are the energy core of cardiomyocytes, and mitophagy is an indispensable mitochondrial quality control mechanism for maintaining cardiac homeostasis [46, 47]. Impaired mitophagy has been implicated in various cardiac diseases [48], but its role in PFAS‐induced cardiac senescence has not been previously established. Our study demonstrates that PINK1/Parkin‐mediated mitophagy is a critical downstream target of the CDK4/MERCs axis in PFOA/HFPO‐TA cardiotoxicity: MERCs provide the spatial scaffold for PINK1 recruitment to damaged mitochondria, and CDK4 suppression‐induced MERCs disorganization directly abrogates this recruitment, leading to the failure of mitophagy and the accumulation of dysfunctional mitochondria. This finding extends the known regulatory role of MERCs in mitophagy to the field of environmental toxicology, highlighting that MERCs are not only a structural link between mitochondria and the endoplasmic reticulum but also a key functional node mediating pollutant‐induced organ damage. Additionally, we found that PFOA/HFPO‐TA exposure inhibits the Sirt3‐SOD2 antioxidant axis, which further exacerbates mitochondrial ROS production and oxidative stress, creating a synergistic effect with mitophagy defect to promote mitochondrial damage and mtDNA leakage [49, 50, 51]. Such synergy underscores the complexity of PFOA/HFPO‐TA‐induced cardiac toxicity and the importance of targeting the entire mitochondrial quality‐control system for intervention.

As a classic detector of cytosolic DNA, the cGAS‐STING axis orchestrates innate immunity [52]. Aberrant activation of this signaling cascade has recently been implicated in the onset of cellular senescence across multiple disease settings [53]. However, its involvement in PFAS‐induced cardiac senescence remains unreported. Our study shows that mtDNA leakage from damaged mitochondria is the primary trigger for cGAS‐STING pathway activation in PFOA/HFPO‐TA‐exposed cardiomyocytes, and this cGAS‐STING signaling is a critical downstream event driving cardiac senescence. Furthermore, we performed causal validation using cGAS knockdown and STING pharmacological inhibition in vitro. Both interventions significantly attenuated PFOA/HFPO‐TA‑induced upregulation of p16/p21, SASP factors, and SA‑β‑gal positivity, demonstrating that the cGAS‑STING pathway is required for the observed senescent phenotype. These findings bridge the gap between mitochondrial dysfunction and cellular senescence in PFOA/HFPO‐TA cardiotoxicity, revealing a novel mechanistic cascade: PFOA/HFPO‐TA‑induced mitophagy defect leads to mtDNA leakage into the cytosol, which in turn activates the cGAS‑STING pathway and ultimately culminates in cardiac senescence. Moreover, the more robust activation of this pathway in the HFPO‐TA group further explains the enhanced senescence phenotype induced by HFPO‐TA, confirming that the cGAS‐STING pathway is a key mediator of the differential toxicity of PFOA and HFPO‐TA.

CDK4 is well characterized as a regulator of G1→S cell cycle progression, and its inhibition by p16/p21 is a core molecular event of cellular senescence [54, 55, 56, 57, 58]. Emerging studies have uncovered non‐cell‐cycle functions of CDK4, including its role in regulating MERCs structural stability [59, 60, 61, 62], but its involvement in environmental pollutant‐induced organ senescence has not been explored. Our study is the first to report that CDK4 is a central hub connecting PFOA/HFPO‐TA exposure, MERCs dysfunction, mitophagy defect, and cardiac senescence. To directly test the causal role of CDK4, we performed CDK4 overexpression rescue experiments. CDK4 overexpression significantly restored MERCs integrity, PINK1 mitochondrial recruitment, autophagic flux, and suppressed cGAS‐STING activation and senescence markers. CDK4 suppression is the initial trigger of the entire toxic cascade, and its downregulation is both a cause and a consequence of cardiac senescence. This finding expands the biological function of CDK4 to environmental health science, identifying CDK4 as a key molecular target that links cellular senescence to organelle quality control in the context of pollutant exposure. Furthermore, molecular docking and functional experiments suggest that CAG binds stably to CDK4 and restores its expression in PFOA/HFPO‐TA‐exposed cardiomyocytes, which in turn stabilizes MERCs, rescues PINK1 mitochondrial recruitment, restores mitophagy function, and ultimately inhibits cGAS‐STING activation and cardiac senescence. These data support CDK4 as a key molecular target of CAG. However, given that natural products often exert their effects through multiple pathways, it remains possible that CAG may modulate other senescence‐related signaling cascades beyond CDK4. Future studies will be necessary to determine the relative contribution of CDK4‐dependent versus CDK4‐independent mechanisms to the anti‐senescence effects of CAG. Nevertheless, CAG exhibits protective effects against PFAS‐induced cardiac senescence at the cellular level, making it a promising natural product for mitigating cardiac damage and represents a candidate for further evaluation.

This study has important implications for environmental health risk assessment and regulatory policy of PFAS. For decades, the toxicological research of PFAS has focused primarily on hepatotoxicity, and HFPO‐TA has been widely used as an industrial alternative to PFOA following global regulatory restrictions on PFOA. However, this assumption of “fewer toxic alternatives” has been challenged by new toxicological evidence. Our findings demonstrate that HFPO‐TA has stronger cardiac toxicity than PFOA, challenging the current one‐sided risk assessment of PFAS replacements that only considers hepatic toxicity. This indicates that the environmental health risk assessment system of PFAS needs to be improved to include cardiovascular toxicity as a key evaluation index, and the environmental exposure limit of HFPO‐TA should be re‐evaluated based on its multi‐organ toxic effects. Additionally, CDK4 is identified as a potential molecular biomarker for PFAS‐associated cardiovascular damage: its downregulation level is closely correlated with the severity of cardiac senescence, which can be used for health screening and risk stratification of populations in PFAS‐contaminated areas. CAG, as a bioactive component of *Astragalus membranaceus*, a traditional Chinese medicinal herb, with low toxicity and high bioactivity, has great potential for development as a preventive intervention agent for these populations, providing a novel natural product‐based strategy for mitigating pollutant‐induced cardiac senescence. Further pharmacological studies are required to define the target specificity of CAG and to determine whether its anti‐senescence effects are mediated exclusively through CDK4.

This study also has several limitations that warrant consideration in future research. First, the doses used here exceed typical human exposure levels, and further studies are needed to verify the toxic effects of environmentally relevant low doses and explore the long‐term cumulative toxicity of chronic low‐dose exposure, which will improve the human extrapolation of our results. Second, the protective effect of CAG was only verified at the cellular level, in vivo studies are needed to optimize its administration dose, route, and pharmacokinetic characteristics, and to evaluate its long‐term protective effect against PFAS‐induced cardiac senescence. Third, we used Baf A1 functional blockade to demonstrate that impaired autophagic flux contributes to senescence. Future studies will perform dynamic mitophagy assays such as mt‐Keima live‐cell imaging to directly visualize mitophagic flux. Fourth, only male mice were used. Future studies will include female mice to investigate potential sex differences in PFAS‐induced cardiac senescence.

## 4 Conclusion

In conclusion, the present study demonstrates that PFOA and its industrial replacement HFPO‐TA induce cardiac senescence through the CDK4/MERCs/PINK1 axis. PFOA/HFPO‐TA exposure suppresses CDK4 expression, destabilizes MERCs, impairs PINK1 mitochondrial recruitment and mitophagy function, triggers mtDNA leakage and subsequent cGAS‐STING pathway activation, and ultimately drives cardiac senescence, with HFPO‐TA exerting more potent effects. CAG ameliorates PFOA/HFPO‐TA‐induced cardiac senescence in vitro by restoring CDK4 expression, stabilizing MERCs, and rescuing the entire mitophagy‐senescence regulatory network. These findings identify CDK4 as a potential molecular biomarker and therapeutic target for PFAS‐associated cardiovascular toxicity, and highlight CAG as a promising natural product for mitigating pollutant‐induced cardiac senescence. Collectively, this study fills a critical gap in understanding the molecular mechanisms of PFAS‐induced cardiac damage and provides important insights for the comprehensive environmental health risk assessment of PFAS replacements, as well as for the development of effective intervention strategies against pollutant‑induced organ senescence.

## 5 Methods

### 5.1 Animals

Male ICR mice (6 weeks old; Liaoning Changsheng Biotechnology, China) were acclimatized for seven days to controlled housing (22‐24°C; 12 h light/12 h dark cycle; 50–60% humidity) before the study began. Mice were then randomly allocated into three groups (*n* = 10 each): vehicle control, PFOA, and HFPO‐TA. The dose of 7 mg/kg for PFOA was selected based on established subchronic toxicity studies in mice, which falls within the effective range (5–10 mg/kg). This dosage reliably induces metabolic and organ damage without causing acute toxicity after 28 days of oral gavage [63, 64]. HFPO‐TA was administered at 0.7 mg/kg as a comparative dose for toxicity evaluation, consistent with dosage ratios commonly used for PFOA alternative assessments in comparative toxicological studies [65]. Test compounds were delivered by gavage in 200 µL of deionized water; controls received an equal volume of vehicle. On day 29, animals were euthanized under isoflurane anesthesia, and hearts were rapidly excised for subsequent analyses. All experimental procedures and processes in this study were unanimously approved by the Animal Welfare Ethics Committee of Northeast Agricultural University (NEAUEC202403138).

### 5.2 Histopathological Analysis

Upon excision, hearts were fixed in 4% paraformaldehyde, then dehydrated. Subsequently, these heart tissues were prepared into paraffin samples and cut into 5 µm‐thick sections for hematoxylin and eosin (H&E) staining.

### 5.3 SA‐β‐gal Staining

Fresh cardiac tissue was rapidly frozen in liquid nitrogen and then embedded in O.C.T frozen section embedding agent (SAKURA, Japan). Slicing was done using a Leica automatic slicer (Leica, Germany). After washing three times with PBS, staining was performed using SA‐β‐gal staining agent. The operation steps were carried out in accordance with the manufacturer's protocol (MCE, USA). The optical microscope and CaseViewer software (3D HISTECH, Sysmex, Switzerland) were used for inspection and observation.

### 5.4 Ultrastructure Observation

The ultrastructure of hearts was observed using TEM. First, quickly slice the fresh cardiac tissue into 1 mm^3^ cubes and fix them in 4% glutaraldehyde. After rinsing with PBS, the sample was dehydrated through gradient concentrations of ethanol and acetone and embedded, which was used for the subsequent preparation of ultrathin sections.

### 5.5 Cell Culture and Treatment

The HL‐1 cells that have been preserved in the laboratory were cultured in DMEM (MeilunBio, China), with the addition of 10% FBS (Cellmax, China) and 1% penicillin‐streptomycin (Biochannel, China) and were cultivated under the conditions of 37°C and 5% CO2. After the HL‐1 cells adhered and reached 80% confluence, they were incubated with PFOA or HFPO‐TA. The culture medium contained no more than 0.1% DMSO. For cellular stimulation, HL‐1 cells were treated with different reagents, including 20 nM Baf A1 and 1 µM H‐151 (MCE, USA). These conditions were selected based on preliminary cytotoxicity assessments and published literature to ensure effective pathway modulation without inducing nonspecific cell death [66, 67].

### 5.6 Cell Transfection

The CDK4 overexpression plasmid (Seven Biotech, China) and Lipofectamine 3000 (Invitrogen, USA) were each diluted in Opti‑MEM, then combined to form transfection complexes, which were added to HL‑1 cells. An empty pcDNA3.1 vector served as the negative control.

The siRNA targeting cGAS sequence (GAUUGAAACGCAAAGAUAU) (Seven Biotech, China) and Lipofectamine 3000 (Invitrogen, USA) were mixed to form transfection complexes, followed by transfection into HL‐1 cells. In addition, siNC served as siRNA negative control.

### 5.7 Cell Viability Assay

CCK‑8 kit (APExBIO, Houston, USA) was employed to quantify cell viability. After treating HL‑1 cells with various concentrations of PFOA, HFPO‑TA, or CAG, we added 10 µL of CCK‑8 solution per well, incubated for 1 h, and measured the optical density at 450 nm with a microplate spectrophotometer (Shanghai, China).

### 5.8 Western Blot Analysis

Protein extraction used RIPA buffer with protease inhibitors. Samples were run on SDS‑PAGE, transferred to NC membranes, blocked with 5% milk, and incubated with primary antibodies overnight followed by secondary antibodies. Antibody information is given in Table S1. Blots were imaged using an Amersham Imager 600 and quantified with Image J.

### 5.9 Cytosolic Fractions Isolation

Cytosolic fractions were isolated using a Cytoplasmic Extraction Kit (Beyotime, China) according to the manufacturer‘s instructions. The resulting cytosolic fraction was used for both qPCR analysis of mitochondrial DNA (mtDNA) and Western blotting to assess purity.

### 5.10 Quantitative Real‐Time PCR Analysis

Total RNA was extracted using TRIzol reagent (Invitrogen, USA) following established protocols [68, 69]. RNA was reverse‐transcribed using an RT‑PCR Kit (TransGen Biotech, China). qRT‑PCR reactions were run on a QuantStudio 5 Real‑Time PCR System (ABI). Specific primer sequences are provided in Table S2. *GAPDH* served as the reference gene for normalization. For mtDNA analysis, total DNA was separately extracted using a commercial DNA extraction kit (TIANGEN, China), and the nuclear gene *Tert* was used as the reference. Three parallel samples were set up for each group.

### 5.11 MitoSOX

Mitochondrial ROS activity was detected with MitoSOX probe (Invitrogen, USA). Fresh frozen heart sections were incubated with the probe at 37 °C for 10 min, washed with HBSS, and then visualized under a fluorescence microscope (Leica, Germany).

### 5.12 Immunofluorescence (IF)

The frozen slices were rewarmed and washed 3 times. Then permeabilized with 0.2% Triton X‐100 for 10 min, washed and blocked at 37°C for 30 min, and incubated with primary antibody for 1 h. After washing with PBS, the secondary antibody was incubated at 37°C for 30 min in the dark. The nuclear area marked by DAPI was blue. For fluorescence of heart tissue, a 10 µm frozen section should be prepared first, washed with PBS for 3 times, and permeabilized with Triton. The other steps are the same as above. Detailed information regarding antibody specifications and working dilutions is provided in Table S1.

### 5.13 ROS and MMP Detection on HL‐1 Cells

Intracellular ROS content was quantified employing a ROS detection kit (MCE, USA). The cells were measured for MMP using JC‐1 fluorescent probe (MCE, USA). According to the kit instructions and then visualized under a fluorescence microscope (Leica, Germany).

### 5.14 Cell Migration Assay

A scratch wound healing assay was employed to evaluate cell migration, observed using an EVOS imaging system (Invitrogen, USA), and ImageJ was used to quantify the migration area [70].

### 5.15 Molecular Docking Analysis

The 3D protein database (PDB) file of CDK4 was retrieved from the RCSB database, and the molecular structure file of the main active component of the compound CAG was obtained from the PubChem database. The ligand preparation and optimization were carried out using PyMOL 2.5 software. After processing, AutoDock Vina was used for molecular docking and binding energy calculation. Finally, the results were imported into PyMOL 2.5 for result visualization.

### 5.16 Statistical Analysis

The software GraphPad Prism (version 8.3) was used for statistical comparison. Data were compared by one‑way analysis of variance (ANOVA) followed by Tukey's post hoc test (^*^, *p* < 0.05; ^**^, *p* < 0.01; ^***^, *p* < 0.001; ^****^, *p* < 0.0001). The *p* value less than 0.05 was considered statistically significant. Fluorescence imaging was quantitatively analyzed using Image J (version 1.8). For in vivo experiments, n represents the number of mice per group. For in vitro experiments, n represents the number of independent biological replicates. At least three biological independent replicates were conducted for every quantitative experiment, and data are presented as the mean ± SD.

## Author Contributions

Nuo‐Wa Li: Performed experiments and Writing – original draft. Jun‐Ze Jiang: Editing and formal analysis. Ying‐Ying Liu: Data curation. Tian‐Tian Zhang: Supervision. Kanwar Kumar Malhi: Visualization. Xin Yao and Jin‐Long Li: Project administration and funding acquisition.

## Conflicts of Interest

The authors declare no conflicts of interest.

## Supporting information




**Supporting File**: advs77280‐sup‐0001‐SuppMat.docx.

## Data Availability

The data that support the findings of this study are available from the corresponding author upon reasonable request.
