# Supplementary material for: CDK4/MERCs/PINK1 Axis Drives PFOA/HFPO‐TA‐Induced Cardiac Senescence via Mitophagy Defect and cGAS‐STING Activation: In Vitro Amelioration by Cycloastragenol
Source: Adv Sci (Weinh). 2026 Aug 18;13(56):e77280. doi: 10.1002/advs.77280 (PMC13482932; doi:10.1002/advs.77280)
Supplement: Supplementary file 1 [file ADVS-13-e77280-s001.docx]

**Supporting Information**

**CDK4/MERCs/PINK1 Axis Drives PFOA/HFPO-TA-Induced Cardiac Senescence via Mitophagy Defect and cGAS-STING Activation: In Vitro Amelioration by Cycloastragenol**

*Nuo-Wa Li, Jun-Ze Jiang, Ying-Ying Liu, Tian-Tian Zhang, Kanwar Kumar Malhi, Xin Yao*, Jin-Long Li**

N. Li^1^, J. Jiang^1^, Y. Liu^1^, T. Zhang^1^, K. Malhi^1^, X. Yao^1^, J. Li^1, 2, 3^

^1^ College of Veterinary Medicine, Northeast Agricultural University, 600 Changjiang Street Xiangfang District, Harbin 150030, P.R. China

^2^ Key Laboratory of Diagnosis and Treatment for Epizootic Diseases of Animals in Cold Regions of Heilongjiang Province, Northeast Agricultural University, 600 Changjiang Street Xiangfang District, Harbin 150030, P.R. China

^3^ Key Laboratory of the Provincial Education Department of Heilongjiang for Common Animal Disease Prevention and Treatment, Northeast Agricultural University, 600 Changjiang Street Xiangfang District, Harbin 150030, P.R. China

*Correspondence: Xin Yao (yaoxin850682712@outlook.com); Jin-Long Li (Jinlongli@neau.edu.cn).

**Figure S1. Validation of cytosolic fraction purity.**

**Figure S2. CCK-8 assay.**

**Figure S3. SASP factors in HL-1 cells.** Data are mean ± SD. n = 3. **P* < 0.05, ***P* < 0.01, ****P* < 0.001, *****P* < 0.0001.

**Figure S4. CAG inhibited PFOA/ HFPO-TA-induced cell damage in HL-1 cells.** (A) Cell migration ability; Remaining scratch area; (B) Microscopic observation was made after culture; (C) ROS level. Data are mean ± SD. n = 3. **P* < 0.05, ***P* < 0.01, ****P* < 0.001.

**Table S1. Antibodies used in this study for Western blot and immunofluorescence staining**

| **Antibody name** | **Company** | **Product category** | **Dilution ratio** |
| --- | --- | --- | --- |
| Anti-p21 | Abclonal | A1483 | 1:1000/1:400 |
| Anti-p16 | Abclonal | A0262 | 1:2000/1:400 |
| Anti-cTNT | Proteintech | 68300-1-Ig | 1:400 |
| Anti-Beclin 1 | Abclonal | A17028 | 1:1000 |
| Anti-p62 | Proteintech | 18420-1-AP | 1:6000 |
| Anti-LC3B | Abmart | T55992S | 1:2000 |
| Anti-Parkin | Affinity | AF0235 | 1:1000/1:200 |
| Anti-Sirt3 | Bioss | bs-6105R | 1:1000 |
| Anti-PINK1 | Abclonal | A25177 | 1:1000/1:200 |
| Anti-SOD2 | Abclonal | A19576 | 1:10000 |
| Anti-IMMT | Abclonal | A2751 | 1:1000 |
| Anti-ATAD3A | Abclonal | A8230 | 1:1000 |
| Anti-SAMM50 | Abclonal | A3401 | 1:1000 |
| Anti-CHCHD3 | Abclonal | A8584 | 1:1000 |
| Anti-TFAM | Abclonal | A3173 | 1:5000 |
| Anti-PERK | Affinity | AF5304 | 1:1000 |
| Anti-Phospho-PERK | Abclonal | AP1501 | 1:1000 |
| Anti-cGAS | Abclonal | A8335 | 1:1000 |
| Anti-elF2α | Abclonal | A21221 | 1:4000 |
| Anti-Phospho-elF2α | Abclonal | AP0692 | 1:4000 |
| Anti-STING | Abclonal | A20175 | 1:1000 |
| Anti-CDK4 | Abclonal | A0366 | 1:1000/1:200 |
| Anti-p53 | Bioss | bs-8687R | 1:1000 |
| Anti-p-p53 | Bioss | bs-3703R | 1:1000 |
| Anti-FUNDC1 | Abclonal | A16318 | 1:1000 |
| Anti-IP3R | Abclonal | A4436 | 1:1000 |
| Anti-MFN2 | Abclonal | A19678 | 1:1000 |
| Anti-TOM20 | Proteintech | 11802-1-AP | 1:400 |
| Anti-Calnexin | Proteintech | 66903-1-Ig | 1:400 |
| HRP-conjugated secondary antibody- | Bioss | bs-40295G-HRP | 1:3000 |
| Goat Anti-Rabbit IgG H&L (Alexa Fluor 594) | Abcam | ab150080 | 1:500 |
| Goat Anti-Rabbit IgG H&L (Alexa Fluor 488) | Beyotime | A0423 | 1:200 |
| Goat Anti-Mouse IgG H&L (Alexa Fluor 594) | Proteintech | SA00013-3 | 1:200 |

**Table S2. Sequences of oligonucleotide primers for qPCR and qRT-PCR.**

| **Name** | **Sequence (5’-3’)** |
| --- | --- |
| ND1-F | TCCCCTACCAATACCACACCC |
| ND1-R | GCTACGGCTCGTAAAGCTCC |
| COX1-F | GCCCCAGATATAGCATTCCC |
| COX1-R | GTTCATCCTGTTCCTGCTCC |
| ND2-F | ATAGGGGCATGAGGAGGACT |
| ND2-R | TGAGTAGAGTGAGGGATGGGTT |
| Tert-F | TCTACCGCACTTTGGTTGCC |
| Tert-R | CAGCACGTTTCTCTCGTTGC |
| ANP-F | CAGATCTGATGGATTTCAAGAACCT |
| ANP-R | GCTTCCTCAGTCTGCTCACTCA |
| BNP-F | GAGCAATTCAAGATGCAGAAGCT |
| BNP-R | GTGAGGCCTTGGTCCTTCAA |
| β-MHC-F | TTACTTGCTACCCTCAGGTGG |
| β-MHC-R | CTCCTTCTCAGACTTCCGCA |
| SERCA2α-F | CCTTCTACCAGCTGAGTCATTT |
| SERCA2α-R | CAGATGGAGCCCACGAGCCA |
| TNF-α-F | AAGCCTGTAGCCCACGTCGTA |
| TNF-α-R | GGCACCACTAGTTGGTTGTCTTTG |
| CCL2-F | GCTACAAGAGGATCACCAGCAG |
| CCL2-R | GTCTGGACCCATTCCTTCTTGG |
| IL-6-F | TCTAATTCATATCTTCAACCAAGAGGTAA |
| IL-6-R | GAATTGGATGGTCTTGGTCCTTA |
| IL-1β-F | ATGCCACCTTTTGACAGTGATG |
| IL-1β-R | TGATGTGCTGCTGCGAGATT |
| GAPDH-F | TCGGAGTCAACGGATTTGGT |
| GAPDH-R | TTCCCGTTCTCAGCCTTGAC |
